# Healthcare trajectories of aging individuals during their last year of life: application of process mining methods to administrative health databases

**DOI:** 10.1186/s12911-025-02898-9

**Published:** 2025-02-05

**Authors:** Delphine Bosson-Rieutort, Alexandra Langford-Avelar, Juliette Duc, Benjamin Dalmas

**Affiliations:** 1 Département de gestion, évaluation et politiques de santé, École de santé publique de l’Université de Montréal (ESPUM), Montréal, Québec Canada; 2 Centre de recherche en santé publique (CReSP), Université de Montréal and Centre intégré universitaire de santé et de services sociaux du Centre-Sud-de-l’Île-de-Montréal, Montréal, Québec Canada; 3 Centre interuniversitaire de recherche en analyse des organisations (CIRANO), Montréal, Québec Canada; 4 Direction de la qualité, de l’évaluation, de la performance et de l’éthique (DQEPE), CIUSSS de l’Ouest-de-l’île de Montréal, Montréal, Québec Canada; 5 Computer Research Institute of Montreal (CRIM), Montréal, Québec Canada

**Keywords:** Process mining, Administrative health data, Event log, End-of-life, Healthcare utilization, Trajectories, Terminal disease, Organic failure, Physical or cognitive frailty

## Abstract

**Context:**

World is aging and the prevalence of chronic diseases is raising with age, increasing financial strain on organizations but also affecting patients’ quality of life until death. Research on healthcare trajectories has gained importance, as it can help anticipate patients’ needs and optimize service organization. In an overburdened system, it is essential to develop automated methods based on comprehensive and reliable and already available data to model and predict healthcare trajectories and future utilization. Process mining, a family of process management and data science techniques used to derive insights from the data generated by a process, can be a solid candidate to provide a useful tool to support decision-making.

**Objective:**

We aimed to *(1)* identify the healthcare baseline trajectories during the last year of life, *(2)* identify the differences in trajectories according to medical condition, and *(3)* identify adequate settings to provide a useful output.

**Methods:**

We applied process mining techniques on a retrospective longitudinal cohort of 21,255 individuals who died between April 1, 2014, and March 31, 2018, and were at least 66 years or older at death. We used 6 different administrative health databases (emergency visit, hospitalisation, homecare, medical consultation, death register and administrative), to model individuals’ healthcare trajectories during their last year of life.

**Results:**

Three main trajectories of healthcare utilization were highlighted: *(i)* mainly accommodating a long-term care center; *(ii)* services provided by local community centers in combination with a high proportion of medical consultations and acute care (emergency and hospital); and *(iii)* combination of consultations, emergency visits and hospitalization with no other management by local community centers or LTCs. Stratifying according to the cause of death highlighted that LTC accommodation was preponderant for individuals who died of physical and cognitive frailty. Conversely, services offered by local community centers were more prevalent among individuals who died of a terminal illness. This difference is potentially related to the access to and use of palliative care at the end-of-life, especially home palliative care implementation.

**Conclusion:**

Despite some limitations related to data and visual limitations, process mining seems to be a method that is both relevant and simple to implement. It provides a visual representation of the processes recorded in various health system databases and allows for the visualization of the different trajectories of healthcare utilization.

**Supplementary Information:**

The online version contains supplementary material available at 10.1186/s12911-025-02898-9.

## Background

The world is aging, leading to a major demographic transition. In 2050, 26.7% of the population of OECD countries is expected to be aged 65 and over [1]. In Canada, these percentages will reach 21.4 to 23.4% by 2030 according to the Statistics Canada scenarios [2]. In addition, the prevalence of multiple chronic diseases has also been increasing for several years [3, 4] and increases with age, from 30% for 40–49 year olds to 52% for 60–64 years old year olds [5]. Therefore, aging individuals may face multiple health problems, leading to increased and more complicated use of healthcare services until their death. Indeed, people at the end of their life utilize a considerable amount of acute healthcare services [6–8]. For several years, the concept of “complexity” has emerged in the attempt to describe the needs of these individuals, but the definition of this concept varies depending on one’s perspective [9–11]. Increasing evidence shows that, from a patient point-of-view, “complexity” is not only a function of the accumulation of medical conditions, healthcare needs or use (e.g., high-level users) but also incorporates organizational, socioeconomic and geographical factors [9–11]. For example, complexity may reflect seeking care from multiple providers or locations or the extent to which their health conditions affect their lives. This complexity leads to significant heterogeneity in the population and its use of healthcare services [12, 13]. This makes adapting the health system to this population even more necessary but also more complex. Indeed, inappropriate use of services is common and can impact the quality of life of individuals already affected by their health condition. This phenomenon has the dual effects of increasing the financial burden on organizations and hampering the quality of life of these individuals. In 2008, Berwick et al.. defined a “triple aim” as: improving individual care, improving population health, and reducing per capita healthcare costs [14]. Their work, then updated by Bodenheimer et al. in 2014 to include providers’ work conditions [15], has become a benchmark for health system organizations to provide to their populations the best care, at the appropriate time and at the best cost. However, population aging, as well as the increase in the number of chronic illnesses, has increased the complexity of providing adequate care to individuals and unbalanced the distribution of the necessary resources. It is therefore necessary to provide more evidence about utilization across the health system to help improve individual care.

Since the early 2000s, research on healthcare trajectories has gained importance, as such trajectories could provide more in-depth information about utilization, help anticipate patients’ needs and better organize services to maximize the value and quality of provided care [16, 17]. Although the term “trajectories” has since been widely used and can refer to different concepts, as depicted by a systematic review performed by Pinaire et al. [18], it relies primarily on patient disease or predicted disease evolution [16, 17, 19]. Indeed, the implementation of “patient trajectories” in the form of a sequence was a step toward more advanced methodologies, making it possible to indicate whether the trajectories conformed to a recommended guideline for chronic illness management or surgery follow-up or even an expected disease evolution [17, 20–23]. However, they are mostly done “by hand”, step by step, through interviews with professionals to capture their essence and formalize it. Unfortunately, models built in this way can be relatively disconnected from reality and difficult to generalize, as they propose an idealized version of reality. Moreover, this modelling exercise is time consuming and requires much expertise. In a context where management is frequently evolving and providers or experts have limited time to allocate to these studies, it is necessary to seek methods that are automated and based on reliable data.

Process mining is a family of techniques emerging from data science that provide information about a process through the data generated by that same process, combining both process management techniques (business process and operations research) and data science (e.g., data mining, predictive analytics, etc.) [24]. This versatile method can be applied in any industry that collects data from its processes; therefore, the healthcare system is a perfect domain for this method since data are generated at every system‒user interaction. For example, a recent study demonstrated the potential of process mining to model the trajectories of patients with amyotrophic lateral sclerosis and provided an overview of the evolution scenarios of this population [25]. Another study from Winter and al. showed than process mining could complement other data-driven techniques and improve our potential to analyze longitudinal or dynamic data [26]. As it relies on event logs, i.e., the collection of all events occurring within a process, including the activity name and time of execution for each event, this “reality analysis” could enable healthcare systems to achieve the “quadruple aim” [15, 24]. On the basis of the way it operates, process mining methods present several benefits over classical descriptive analysis or even machine learning methods. Indeed, when administrative health databases are used, it is relatively easy to aggregate knowledge about healthcare use and compute quick indicators to display on dashboards, such as *“80% of patients had an appointment with a physician”* or *“only 30% of patients visited the emergency department”* (ED). Although these indicators make it possible to have an overall view, they are too limited to allow an understanding of the different patients’ journeys. *Are the 30% of patients who visited the ED part of the 80% who consulted a physician? If so*,* did they consult before or after their ED visit?* The answers to these questions can be obtained via more detailed stratified analysis but involve long and tedious analysis to perform all possible combinations of services and temporality. Machine learning methods, such as unsupervised clustering, are a step toward a better assessment of healthcare trajectories, as they can be used to identify characteristic patterns of healthcare utilization, regardless of user knowledge or medical status. However, this trajectory formalism remains “linear” and “macro”. It can provide visualization of patients’ journey as a temporal sequence and offers the possibility of visualizing and characterizing consumer groups but does not allow for easy presentation of the variations or “deviations” experienced by certain (and potentially rare) patients. Indeed, as in many cases, the Pareto principle applies, and 80% of the “issues” (patient dissatisfaction, high costs, medical referral errors, medical care delays, etc.) are often related to 20% of patients. Thus, the simple trajectory method is limited in highlighting the “rare” or less frequent trajectories that may cause the most unnecessary resource consumption. Additionally, this presentation of trajectories in a temporal sequence form can be difficult to use directly for informed decision making since it requires important analysis and interpretation effort, as it would be necessary to study all trajectories to assess compliance with the overall (or average) trajectory or a recommended guideline.

From this perspective, process mining represents a real opportunity to provide a methodology to represent the reality of the field, its bottlenecks, deviations and “unnecessary” resource consumption, while providing a readable tool for decision making. In addition to the statistics of care and the main trajectory that can be found in the model, process mining provides a complete map of all the patient paths actually identified in the field. This map can be used for different purposes, such as *(i)* to represent the global patients’ trajectories, *(ii)* to analyze certain elements of the process, such as undesired deviations, *(iii)* to highlight elements of the process on which it would be necessary to perform a field investigation (bottleneck, etc.), and finally *(iv)* to allow the appropriate questions to be asked: *Why did 30% of patients visit the emergency room? Why did half of them never receive any follow-up? Why are there so many readmissions after a hospital stay? All the people admitted to the LTC saw their family doctor beforehand; is this a mandatory condition for admission?* The various filtering and stratification possibilities of the method make it a very serious candidate for accurately modelling patients’ trajectories.

However, several challenges remain in order to model quality health trajectories. Currently, most process discovery applications focus on intraorganizational processes, i.e., process execution within a single organization. In the context of healthcare trajectories, patients’ interactions need to be captured across several organizations. Therefore, a challenge arises since we need to consider different information systems with different characteristics (e.g., granularity, quality, representativeness, etc.). Our objective was therefore to conceptualize this care trajectory logic and model it via a process mining method. Process mining was applied to administrative health data collected by the healthcare system for each user’s utilization of healthcare services during their last year of life.

This general objective was divided into clinical and methodological subobjectives, namely, *(1)* identifying the healthcare baseline trajectories during the last year of life, *(2)* identifying the differences in trajectories according to medical condition, and *(3)* identifying adequate settings to construct a quality event log and provide a readable map via administrative health databases.

## Methods

**Fig. 1:**
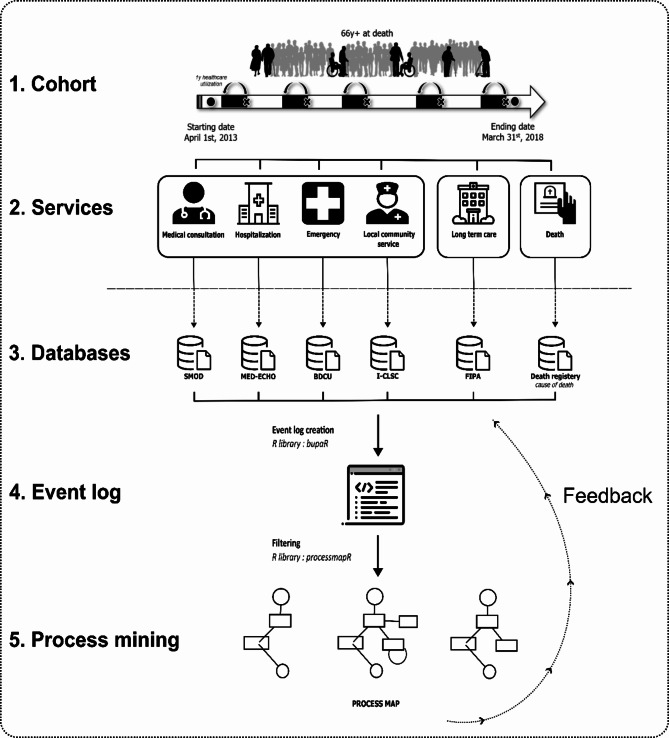
Diagram of population, data sources and modelling (**1**) Study population and study period; (**2**) Services utilized by individuals in the cohort; (**3**) Databases recording healthcare utilization by service type; (**4**) Transformation of data into an event log; (**5**) Modelling of process map based on the event log

A retrospective quantitative longitudinal research study design was used to analyze individuals’ healthcare trajectories during their last year of life. Administrative and administrative health data were provided by the *Régie de l’assurance maladie du Québec* (RAMQ) and the Ministry of Health (MSSS) through a tripartite agreement with the National Institute for Excellence in Health and Social Services (INESSS) in Quebec (see declaration of authors). Data were anonymized, with a unique anonymized identifier for each individual, enabling the linkage of the databases. All data management and analysis were performed using R version 4.1.3 [27]. Process mining was applied using the *bupaR* library [28].The study population, data sources and modelling process are illustrated in Fig. 1.

### Study population and study period

The population source consisted of individuals who died between April 1, 2014, and March 31, 2018, and were at least 66 years or older at death (Fig. 1.1). As we aimed to study the last year of life of healthcare utilization (Fig. 1.2), the 66-year-old threshold was established to increase our ability to retrieve healthcare and service use within administrative health databases. Indeed, Quebec drug insurance is offered free of charge to seniors aged 65 years and over. The total study period covered all healthcare utilization from April 1, 2013, to March 31, 2018, and was established in accordance with the most recent and complete data available at the time of the project. A representative sample of 10% was extracted and corresponds to the final study population.

### Individual characteristics data

Personal sociodemographic information and causes of death were obtained from the Insured Persons Registration File (FIPA) and death registry (RED/D), which was fed mainly by the death certificate (SP-3) (Fig. 1.3). The date of death was obtained from the death register, and the date index was set as 365 days prior to this date to retrieve all healthcare utilization data one-year prior to death. Age was calculated according to the date of birth and date of death and categorized as follows: 66–69 years, 70–79 years, 80–89 years and 90 years and over. Causes of death were reported via the International Classification of Diseases, 10th edition (ICD-10) and classified according to 5 clinical causes of death adapted from Lunney et al. [16]: terminal illness, organ failure, physical or cognitive frailty, other, and unknown (Table 1). The list of ICD-10 codes included in each category is available in the supplemental file (Supplemental 1). An individual could only be in one category.

**Table 1:** Classification of causes of death according to clinical trajectories

Cause of death (ICD-10 label)	ICD-10 code	Cause of death*
Neoplasms	C00-D48	Terminal illness
Certain infectious and parasitic diseases	A00-B99	Organic failure
Diseases of the blood and blood-forming organs and certain disorders involving the immune mechanism	D50-D89	
Endocrine, nutritional and metabolic diseases	E00-E90	
Diseases of the circulatory system	I00-I99	
Diseases of the respiratory system	J00-J99	
Diseases of the digestive system	K00-K933	
Diseases of the skin and subcutaneous tissue	L00-L99	
Diseases of the musculoskeletal system and connective tissue	M00-M99	
Diseases of the genitourinary system	N00-N99	
Congenital malformations, deformations and chromosomal abnormalities	Q00-Q99	
Mental and behavioral disorders	F00-F99	Physical or cognitive frailty
Diseases of the nervous system	G00-G99	
Diseases of the eye and adnexa	H00-H59	
Diseases of the ear and mastoid process	H60-H95	
Symptoms, signs and abnormal clinical and laboratory findings, not elsewhere classified	R00-R99	Other
Injury, poisoning and certain other consequences of external causes	S00-T98	
Codes for special purposes	U00-U99	
External causes of morbidity and mortality	V01-Y98	
Factors influencing health status and contact with health services	Z00-Z99	

* From Lunney et al. [16]

### Healthcare services data

Healthcare utilization anonymized data were obtained from 6 separate databases, one for each service (Fig. 1.3). Each database contained information related to the use of a specific service by an individual, including the facility visited, the service date or start and end dates when applicable, as well as a range of data on the medical status and the service provided. Information on hospital stay was obtained from the hospitalization registry (MED-ECHO). Emergency visits were obtained from the emergency department common databank (BDCU). Physician service information was obtained from the fee-for-service physician claims file (SMOD). A medical visit was defined as all the medical acts from the billing database occurring the same day (date), performed by the same physician (provider number), within the same institution (institution number) for the same individual (individual number) and subject to the same billing request (NCI number). A distinction has been made to differentiate actual medical visits and consultations occurring during an emergency visit or a hospital stay, using both the location and emergency or hospital stay dates and cross-referencing the information with the BDCU and MED-ECHO databases. Services of professionals who are not paid on a fee-for-service basis, such as visiting hours, some home care services and the vast majority of care provided in the context of palliative care, are not included in this database. Information on clinical interventions by various healthcare providers in local community service centers (CLSCs) was obtained from the CLSC integration file (i-CLSC). As there is no specific database to document LTC accommodations, the current practice in Quebec is to use the insurance plan information provided by the Insured Persons Registration File (FIPA). For each individual, the starting and ending dates of insurance plans 97 (Person accommodated) and 50 (Person eligible for the purchase program) were used to define LTC accommodation periods. Individuals for whom the entry date was prior to the index date (1 year before the individual’s death) were assigned the index date as the accommodation starting date (maximum 365 days).

Using the unique anonymized identifier, all the data were formatted and compiled into a single dataset where each observation corresponded to the unique use of a specific service– hospitalization, emergency visit, medical consultation, long-term care, or community services–by a single individual on a given date. The “*activity*” variable used for process mining modelling corresponded to the type of service provided (e.g. hospitalization, emergency visit). The *timestamp* variable (time of execution) corresponded to the date of service. Individuals could have multiple observations in the final dataset if they utilized services multiples times. For “aesthetic” reasons, a “death” activity was added to the dataset, with the date of death serving as the timestamp. Services recorded postmortem were excluded to ensure model quality.

### Analysis and application of process mining

As described previously, process mining is a versatile family of methods that can be applied in any industry to collect data from its processes. Therefore, the healthcare system is a perfect domain for this method since data are generated during every interaction between the system and users. Process mining allows one to model a process—in this case, healthcare trajectories—and its different deviations through the processing of an event log. An event log is a process-oriented dataset in which registered information related to the different executions of the process under study is used. In the context of our work, the final dataset previously described was transformed into an event log containing detailed information related to the patients and their interactions with the healthcare system, i.e., the “activities” (services) and the timestamp (the time of use) (Fig. 1.4). Information about time, resources, cost and so on can also be added to the model. However, as our objective was to adapt the method to model trajectories over space (e.g., services, organizations, regions) and time (one year), we used the minimal setting to model trajectories, only using the activity and date. To model patient-centric trajectories, we used “case-based” modelling rather than “event-based” modelling (individual users vs. events). The main output of process mining is a process map, which refers to a graph where each activity is represented as a node, and the different paths between activities are represented by edges (Fig. 1.5). In other words, all individuals start at a “starting node” and distribute themselves over the different activities (services) provided in the event log (i.e., hospital stays, emergency visits, medical consultations, interventions by local centers, LTC accommodations, and deaths) according to their own paths of use and end at the ending node. The edges symbolize the transition from one activity to another (succession relationship), indicating to the reader which activities follow each other according to the activity log. The descriptive statistics presented on these edges indicate how many individuals have passed from service A to service B among the individuals who used service A, as well as the average delay between these activities. In the case of consecutive repeated activities, such as emergency or hospital readmission, a loop can be represented by an edge starting and ending with the same activity. Particular attention is needed, as the notion of “consecutive” depends on the granularity of the information integrated into the event log. The “readmission” can then correspond to an actual readmission with a very short time between them or two distinguished admissions spaced over time, implying that there is no other contact with the health system during this period. This is extremely important when reading and interpreting process maps and is therefore important for understanding the granularity of one data point.

### Ethics and data access

This study was approved by the Research Ethics Committee in Science and Health at the Université de Montréal (CERSES 2024–6300). Data access was granted under strict confidentiality agreements by the INESSS institute through its tripartite agreement with the health insurer, Régie de l’Assurance Maladie du Québec (RAMQ), and the Ministry of Health and Social Services (MSSS). Secure access to the data was provided through a VPN within INESSS facility.

## Results

### Characteristics of the study population and healthcare utilization

The merged dataset was composed of 556,742 healthcare utilization (activities) entries for a total of 21,255 individuals within the cohort. A total of 66.7% of the entries were from the fee-for-service database, 16.1% were from the i-CLSC database and related to local community center services, 9.4% were from the emergency database, 6% were from the hospitalization database, and finally, 1.8% were related to LTC accommodations.

Among the 21,255 individuals in the cohort, 11,184 were women (52.6%), whose average age at death was significantly greater than that of men (84.9 and 81.2 years, respectively; *p* < 2.2e-16) (Table 2). The number of deaths per year was stable over time throughout the study. Approximately 40% of individuals who died between 2014 and 2015 and 2017–2018 were aged 80–89 years (all combined). However, nearly 60% of individuals who died between 66 and 69 and 70–79 years of age were males, and 70% of individuals who died at 90 years and over were females. Nearly 50% of the individuals in the cohort died of organ failure (see Table 1 for definition), and 53% of them were women. The largest observable difference between men and women was death due to physical or cognitive frailty, for which nearly 63% were women (Table 2). This is consistent with the fact that more than 80% of deaths in this category were distributed in the 80–89 and 90 + age groups, the majority of which were women. For the analysis, individuals in the “Other” and “Unknown” trajectories were grouped together to ease the reading. Finally, using the emergency room and hospitalization databases, it was estimated that 9.2% of the individuals died in the emergency room (of whom 91.4% died within 24 h of admission), and 11.8% died during a hospital stay (of whom 12.1% died within 24 h of admission). Notably, of all the individuals who died during a hospital stay, 22.6% were admitted to a palliative care unit.

**Table 2:** Population characteristics of the 21,255 individuals who died between 2013 and 2018 in Quebec at 66 years and over

		Terminal disease	Organic failure	Frailty	Other	Unknown	Overall
		*N* = 6,*302*	*N* = 10,*422*	*N* = 3,*189*	*N* = 786	*N* = 556	*N* = 21,*255*
		*29.60%*	*49%*	*15%*	*3.70%*	*2.60%*	
**Sexe (%)**	Female	46.4	53.1	62.8	54.5	52.7	**52.6**
**Age group at death (%)**							
	66–69	14.3	6.8	3.0	8.8	9.0	**8.6**
	70–79	39.7	23.7	14.9	18.4	27.9	**27.0**
	80–89	35.1	41.2	46.4	38.4	37.8	**40.0**
	90 and over	10.8	28.3	35.7	34.4	25.4	**24.4**
**Fiscal year of death**							
	2014–2015	23.6	25.6	24.5	25.7	21.9	**24.8**
	2015–2016	24.7	23.7	22.5	22.0	20.7	**23.7**
	2016–2017	27.1	24.2	24.9	25.8	27.5	**25.3**
	2017–2018	24.6	26.5	28.0	26.5	29.9	**26.2**

### Healthcare baseline trajectory

**Fig. 2:**
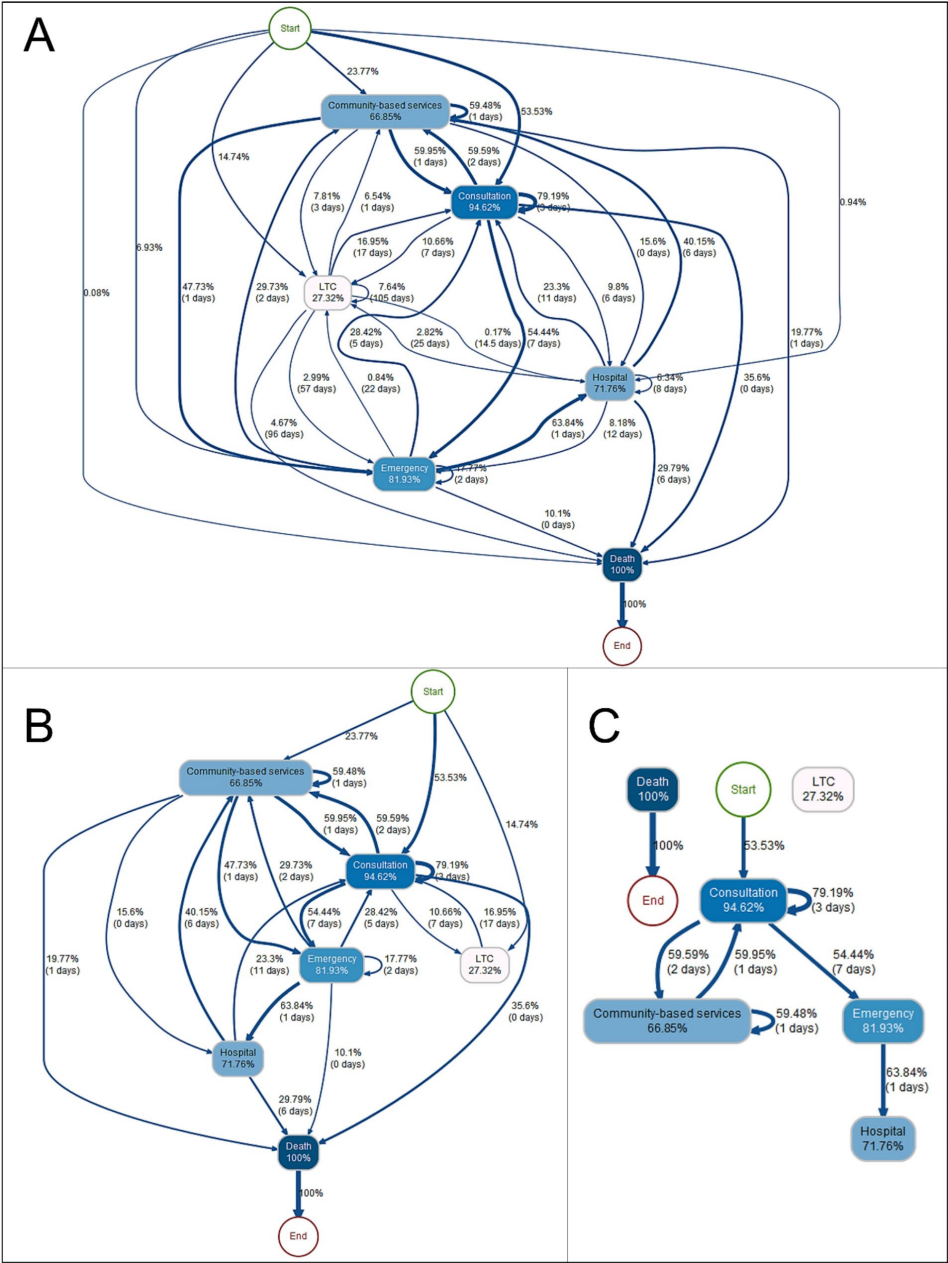
Process maps of healthcare utilization in the last year of life for individuals aged 65 years and over in Quebec between 2013 and 2018. (**A**) General unfiltered map; (**B**) 10% filtered map, only edges followed by a minimum of 10% of the population are displayed; (**C**) For the 50% filtered map, only edges followed by a minimum of 50% of the population are displayed. The node color and edge thickness are proportional to the proportion of individuals involved

To obtain the healthcare baseline trajectory (i.e., the merging of all trajectories), we generated the event log and process map of the 21,255 individuals in the cohort, regardless of age, sex or cause of death (Fig. 2A). The main goal was to identify the baseline trajectory and potential distinct healthcare topologies for further comparisons with stratified process modelling. Additionally, this baseline process map was used to demonstrate the impact of different filter settings applied on the proportion of edges, namely, 10% and 50% (Fig. 2B and C). A 10% filter means that an edge will only appear on the process map between services A and B if at least 10% of the patients who used service activity A subsequently used service B. The median time displayed on the edge refers to the period between the start date of service A and the start date of service B. This median time does not represent the duration of each service (start A– end A), although hospitalization and LTC accommodation durations are automatically covered due to the modelling settings (start A– start B). The node color and edge thickness refer to the proportion of individuals using services and involved in the successional relationships (transition between services).

As expected, the filtering settings can have a relatively important effect on the process map readability and reliability. In Fig. 2A (baseline), 37 edges were identified for a total of 8 nodes, including 5 nodes associated with services, the starting node, the ending node and the additional node indicating death. In Fig. 2B (10% filter), the number of edges was reduced to 29, and we denoted only 14 edges in Fig. 2C (50% filter). As the unfiltered Fig. 2A presents all recorded trajectories, the Fig. 2C illustrates the backbone of these trajectories by showing only the most prevalent successional relationships. However, Fig. 2C masks a very large number of deviant trajectories and can even be confusing in the case of long-term care (LTC) and death nodes, which are graphically isolated from other nodes. In the language of network analysis, an isolated node is a node that fails to be connected to another node. In our specific case, it represents an activity with a very small proportion of individuals coming from or going to several other services and represents a modelling artifact. A process map is a subjective construction, for which the objective is to find a compromise between process variability and cohort representativeness. The 10%-filtered (Fig. 2B) presents an intermediate version of this baseline trajectory, allowing both a simplified reading compared to the unfiltered (Fig. 2A) but more complete than the 50%-filtered (Fig. 2C) in terms of variations and deviations. Empirically, this filtering method also appeared to be effective for stratified modelling and was used for all process map presentations in subsequent analyses. The full-size figures (Fig. 2A-C) are available in the supplemental files.

As noted in the methodology section, the nodes and edges provide the classic volume descriptive statistics (Fig. 2B). It is important to differentiate the nodes’ values from the edges’ values, as the nodes indicate the total number of individuals who benefit from a specific service for the whole period, whereas the edges represent the proportion of people using a specific sequence of services. Below, a process map description serving as a reading grid is provided.

Starting with the nodes, 94.6% of the individuals in the cohort consulted a physician during the last year of life, 81.9% of the individuals were admitted at least once to the emergency room, 71.7% stayed in the hospital, 66.8% benefited from at least one intervention provided by a local community center, and 27.3% of them were housed in LTC. Detailed information about re-entry (consecutive use of the same service) is provided in the loops. Among the 94.6% who consulted a doctor, approximately 79% of them consulted again, with a median time of 3 days between each consecutive consultation. Among the 81.9% of the cohort who visited the ED at least once, 17.7% were readmitted within a median of 2 days. Among the individuals who obtained services from a local community center, 59.5% received them consecutively, with a 1-day median. This repetitive pattern could reflect homecare services, for instance. Interestingly, out of the 71.7% of individuals who stayed in the hospital, 6.3% had a second stay, with a median of 8 days between the two hospitalization starting dates. However, this information was only visible in Fig. 2A, as the filter prevented the display of the edge containing only 6.3% of the population, enhancing the importance of aligning the filter with the decision-makers’ needs.

Looking at the different edges presented in Figs. 2B and 100% of the initial cohort starts from the top (first node) and is then divided according to the first service used at the beginning of the study period, i.e., its last year of life. The beginning of the end-of-life trajectory divided 92.1% of the cohort according to three major edges, highlighting three different patterns of healthcare utilization: 23.8% of the individuals benefited from a local community center intervention, 53.5% began with a medical consultation, and 14.8% of the individuals ultimately started their trajectory in long-term accommodations. For these individuals, it is important to consider potential former admissions, as we reduced LTC accommodations to the study period (a maximum of 365 days). Following this first division, the edges indicate the subsequent services. For example, 54.4% of people visiting a physician then visit the emergency room, with a median delay of 7 days.

To summarize the node/edge information from the local community centers example, from the 66.9% of individuals benefiting from at least one intervention, 23.8% of them directly started their trajectories with this service (15.9% of the users in the cohort), 60% then consulted a physician with a 1-day median delay (40% of the users), 47.7% of them visited the emergency room with an identical median delay (31.9% of users), and 15.6% of them were hospitalized the same day (10.4% of users).

Beyond descriptive statistics, it is interesting to analyze the dynamics between services as well as to reflect upon the real processes that are modeled by these interactions and relationships as well as the data. For example, we observed a certain dynamic between local community center services and medical consultations, with 59.9% and 59.5% of the patients, respectively, having used these two services successively. Moreover, medical consultations seemed to represent a central service with many users (94.6% of the cohort) and strong connections (thick edges) with all the other services, implying that a large part of the population seeing a doctor also uses a wide variety of other services. Similarly, the origin of the individuals in LTC was quite scattered. However, unlike medical visits, the proportions of the cohort using the service and coming from other activities were relatively lower. Indeed, most of the edges actually disappeared due to the filter, and for the 27.3% accommodated in LTC facilities, only two edges were displayed: the origin and medical visits (14.7% and 10.7%, respectively). This could reflect that (1) the precise moment of care cannot be predicted and can therefore follow a set of varied activities or (2) that there is no apparent logic to entering LTC in the last year of life, as this can occur at any point in the trajectory. Notably, this type of interpretation must be made with caution, and various reasons could explain this pattern of utilization. Finally, in a similar fashion, the distribution of various origins for the “death” node indicated that death could occur at any point in the healthcare trajectory. We observed that 29.8% of deaths occurred at the hospital and that 10.1% occurred during an emergency visit. Finally, a total of 35.6% seemed to follow a consultation (median delay of 0 days), which can be partly explained by the fact that death certificates can be established by physicians, in the case of death during an ambulance transfer, for instance, and can therefore be recorded within the billing database.

### Trajectories according to the cause of death

**Fig. 3:**
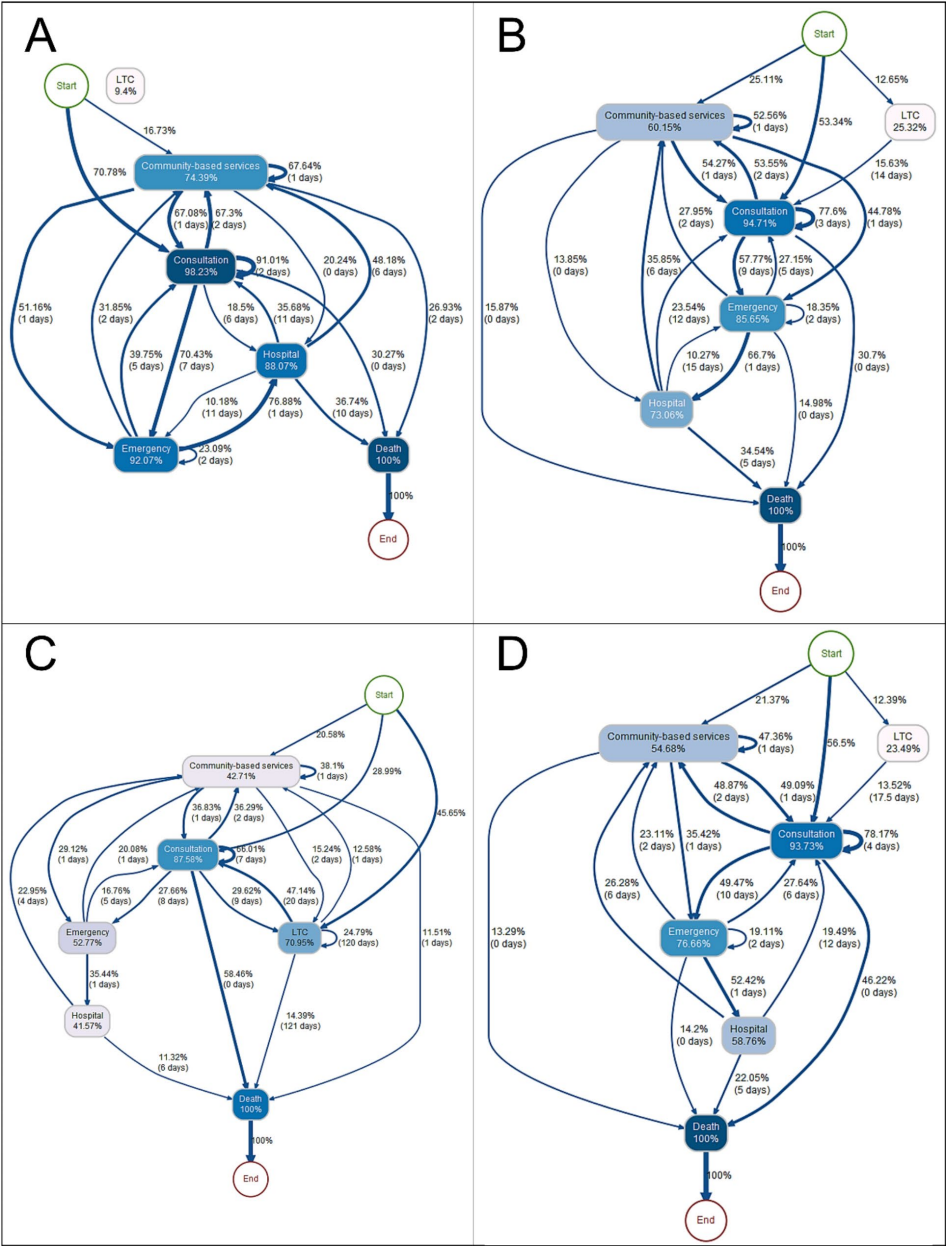
Process maps of healthcare utilization in the last year of life for individuals aged 65 years and over in Quebec between 2013 and 2018 according to their cause of death. (**A**) Terminal disease (10% filtered); (**B**) Organic failure (10% filtered); (**C**) Physical or cognitive frailty (10% filtered); (**D**) Other or unknown (10% filtered). The node color and edge thickness are proportional to the proportion of individuals involved

To demonstrate the potential of process mining and its ability to reflect the real utilization of healthcare services, we modeled the trajectories according to the clinical status (cause of death) Process maps stratified by cause of death and filtered at 10% are presented in Fig. 3. It illustrates the trajectories of the cohort that died from a terminal illness (Fig. 3A), due to organ failure (Fig. 3B), attributable to physical or cognitive frailty (Fig. 3C) and, finally, other or unknown causes (Fig. 3D) The full-size figures (Fig. 3A-D) are available in the supplemental file.

Starting with the healthcare trajectory of the 6,302 individuals who died of a terminal disease (cancer), the first notable observation was the high utilization of all services, except LTC accommodations (Fig. 3A). In that respect, 98.2% of them consulted a physician at least once during their last year of life, 91% of them consulted consecutively and repeatedly (median delay of 2 days), and 70.8% of them had this medical consultation as their first activity. Considering the other services, 92% of the patients visited the emergency department at least once, 88.1% stayed in the hospital, and 74.4% benefited from local community center interventions. We noted an important dynamic between the different services, with important transitions involving, most of the time, at least 30% of the individuals, with the consultation remaining the central service. For example, 76.9% of individuals who had been to the emergency department subsequently stayed in the hospital (median delay of 1 day), and 10% of individuals who had been hospitalized visited the emergency department again after a median delay of 11 days. Another interesting dynamic is the back-and-forth utilization of local community center services and medical consultations, with 67% of individuals having used these two services consecutively, with median delays of 1 or 2 days. Additionally, the large loop on local community center services with a median delay of 1 day (67.6% of the cohort) seems to indicate an intensive follow-up of individuals with a terminal illness who used local community center services. A final interesting element is also the presence of the seemingly isolated node of accommodation in LTC, concerning only 94% of individuals who died of a terminal illness. As a reminder, the isolation of this node only indicates that the proportion of individuals passing through this service from other services is less than 10%.

Considering the specificity of the terminal disease, where a vital prognosis is engaged, it is interesting to compare this trajectory to trajectories of individuals who died of organ failure (Fig. 3B) or who are physically or cognitively frail (Fig. 3C). The distribution of individuals according to the first service occurring during the study period varied greatly according to the different causes of death. While consultation was the first service used by 708% of individuals suffering from a terminal disease, only 53.3% and 29% of individuals who died of organ failure or with physical and cognitive frailty used it first, respectively (Fig. 3B and C). Thus, 25.1% of individuals who died of organ failure began their trajectory with a local community center intervention, and 12.7% appeared to be already living in an LTC facility (Fig. 3B). Among the individuals who died of physical and cognitive frailty, 20.6% initiated their trajectory through the local community center, 45.7% of whom were likely already accommodating (Fig. 3C). Ultimately, while medical visits remained a central service for people suffering from organic failure, with 94.7% of individuals who consulted, this proportion dropped to 87.6% for individuals who were affected by physical or cognitive frailty.

In terms of the relationships between accommodations in LTC centers, services offered by local community centers, and the use of emergency rooms and hospitalizations (Fig. 3A-C), notable differences can be observed. Individuals who died of organ failure were admitted to LTC facilities more often than those with a terminal illness (25.3% and 9.4%, respectively) but were admitted much less often than individuals with physical or cognitive frailty, for whom 71% were housed in LTC facilities during their last year of life. Additionally, 60.2% of individuals who died of organ failure used local community center services, which is lower than that of individuals who died of cancer but much higher than that of individuals suffering from physical and cognitive frailty, with only 42.7% of them using this service. This observation is similar to that of the emergency department (857% for organ failure and 52.8% for physical and cognitive frailty). However, the difference is less important for hospitalization (58.8% and 41.6%, respectively), which indicates that despite regular care provided by local community centers or LTCs, a certain proportion of hospitalizations cannot be prevented. All these elements are consistent with the information provided by expert and healthcare professionals (including palliative care physicians and the Quebec End-of-Life Care Commission), who were consulted during the project. Indeed, individuals who die of a terminal illness generally see an increase in their use of acute services when their vital prognosis is announced due to the implementation of a palliative care procedure. However, individuals suffering from physical and cognitive frailty may live with their disease for several years before dying, sometimes involving a loss of autonomy and requiring daily follow-up in an LTC facility, for example.

As these process maps illustrate, process mining can easily represent the differences in healthcare utilization and health management according to individuals’ health status without having to rely on the computation of multiple indicators and stratification.

## Discussion

Our general objective was to conceptualize the healthcare trajectory and model it via a process mining method. To reach this objective, the subobjectives of this work were manifold. The methodological objective was to assess the feasibility of adapting the process mining methodology to represent patients’ trajectories over multiple services, multiple organizations and several financial years. As this methodology was mostly used to represent a single process, or a set of processes, within a single organization, the extension of its application to a multiorganization and multiregional context over a long period of time represented a methodological and visual challenge. To our knowledge, there is no work that has applied this methodology to model a trajectory over such a vast territory (province of Quebec), implying a plurality of practices and contexts and using 6 different databases (including the i-CLSC database), entailing a variety of data collection and quality due to their different purposes. Additional objectives were to describe the healthcare baseline trajectory during the last year of life of individuals in Quebec who died between 2014 and 2018, as well as the topologies of healthcare and service use observed during the last year of life according to the cause of death. We chose to illustrate the adaptation using the cause of death, as research on trajectories is often based on health status, allowing us to properly compare and validate this method. Moreover, these clinical categories have been used in other provincial studies by both the Quebec National Institute of Public Health (INSPQ) [29, 30] and the National Institute for Excellence in Health and Social Services (INESSS) [31] and allow valid comparisons of our findings.

On the basis of an event log composed of healthcare service utilization, process mining modelling allows visual and intuitive representation of the different topologies of healthcare-consuming individuals during their last year of life. Globally, we observed three main trajectories of healthcare utilization, for which medical consultation was a central service, which seems to be fairly consistent with the fact that physicians are still the core of primary care access [32, 33]. These three main trajectories can be defined as *(i)* mainly accommodating a long-term care center; *(ii)* providing services by local community centers in combination with a high proportion of medical consultations and acute care (emergency and hospital); and *(iii)* combining consultations, emergency visits and hospitalization with no other management by local community centers or LTCs. Although not groundbreaking, these results support the ability of process mining modelling to provide a quick and faithful representation of patients’ trajectories via the use of administrative health data, highlighting specific or deviant patterns. This aspect could be a major advantage for organizations, as they could use their administrative health data to monitor patients and healthcare utilization across their facilities opportunely, going beyond the tunnel vision offered by single and independent indicators presented on a dashboard side-by-side.

Stratifying according to the cause of death highlighted that LTC accommodation was preponderant for individuals who died of physical and cognitive frailty. Conversely, services offered by local community centers were more prevalent among individuals who died of a terminal illness. This difference is potentially related to the access to and use of palliative care at the end-of-life, especially home palliative care implementation. Some authors have shown a difference in access to and use of palliative and end-of-life care between individuals according to their health condition; for example, Seow et al. reported that individuals who died of a terminal illness received up to twice as many days in palliative care as individuals who died of organ failure or physical and cognitive frailty [34]. Interestingly, there is no clear operational definition of access to palliative and end-of-life care in Quebec, although practitioners believe that a vital prognosis of 3 months must be established to provide palliative care. Thus, it is not surprising to note a difference in the use of palliative care at the end-of-life depending on the cause of death, since it is easier to provide a vital prognosis for terminal illness (cancer) than for organ failure or physical and cognitive frailty, the decline of which is much more sudden or much longer before death, respectively. These elements were also raised in a recent report describing home care for individuals who may benefit from palliative care in the province [31]. Therefore, the healthcare trajectories stratified by cause of death seem to appropriately reflect the reality of the healthcare provision at the end-of-life in Quebec. Baseline process maps were also modeled according to the biological sex and age at death (data not shown). However, biological sex basically reflects the cause of death, as we observed more women than men housed in LTC facilities, which was consistent with the higher prevalence of women dying from cognitive or physical frailty, who are the main users of LTCs. Finally, age was equally subject to many confounding factors to be relevant.

On the basis of these realistic results, the potential of process mining to describe healthcare trajectories via administrative health data is promising. This methodology has three main advantages. First, it is relatively easy to model the trajectories on already cleaned data since it basically requires only the name of the activity, the date and the beneficiary to generate an event log that can then be filtered on specific population groups if needed. The remaining methodological difficulties rely more on data cleaning and the definition of the activities to be modeled, which are common to any classic analysis involving administrative health or research data. However, this argument is valuable only for the descriptive aspect of process mining, excluding any predictive process monitoring methods aimed at predicting the future of an ongoing process execution. Typical examples of predictions relate to the execution outcome, the completion time, or the future sequence of steps in the execution [35, 36]. Predictive process monitoring methods have yielded effective results and have been applied to many domains [37, 38]. However, owing to its complexity and high variability, healthcare has always been a real challenge in regard to applying process-oriented analytics methods [39], and process mining and predictive process monitoring are no exception [40]. More work is needed to apply predictive process monitoring to administrative health data. A second advantage is that obtaining an intuitive visualization can allow a more fluid interpretation of the different dynamics that exist between services. This element can be highly important in the context of decision support since the multiplicity of tables and stratified analysis can quickly become a challenge for understanding and interpretation, known since the 2000s as “information overload” [41]. Third, the capacity of the model to represent the reality of field processes and to encourage questioning is a great asset, especially with respect to the organizations’ quadruple aim ambition to provide the best care, at the best time, for the best cost while respecting their workers. These questions can be related not only to the process but also to the collected data and can support reflection to improve not only the organization, highlighting the “hidden process” but also the data quality and collection. This is an important step in moving forward a learning system.

Interestingly, despite the slightly “old” study period (2014–2018), these elements remain relevant for the MSSS and the network. Indeed, the plan for change proposed by the MSSS in April 2022 highlights a desire to offer a “front line of the future” to facilitate access to health services (physician and emergency) and to encourage a shift toward home support for seniors and vulnerable persons [42]. However, there is no clear mention of the desire to promote death at home, which is preferred by individuals in 70% of cases according to an old study dating from 2013 [7]. There is therefore important work to come, and process maps could perhaps constitute one of the key tools allowing both the visual representation of what is “truly happening on the ground” and the genesis of different questions and working hypotheses for improving services and their access when the data are available.

Additionally, we present the trajectories of individuals whose ages vary between 66 and 108 years, which may have an impact on the presentation of trajectories. Considering space, there are both variations in the offer and use of health services according to the territories (e.g., regulations, offer, level of saturation) and variations according to health typology (e.g., illness, dependence). For these reasons, the stratification of process maps according to these major groups may be relevant for better interpretation and added value for decision support.

### Limitations

Despite these advantages, the process mining applied to administrative health data has its limitations. Indeed, using administrative health data always represents a challenge, as these data are not collected for research purposes, which may result in disparities in their quality and reliability. Fortunately, in the case of process mining, the variables of interest are important and mandatory variables for the healthcare itself (database source, timestamp), ensuring strong reliability of the data. However, some limitations remain, either related to the data management choices or the data itself, as the quality can vary depending on the sources or the methodology itself.

#### Administrative health data management

While the emergency visit and hospital stay data are the most reliable, as they are routinely used by the health system for performance assessment, the fee-for-service billing and local community service center databases (SMOD and i-CLSC) required manipulation.

As medical acts from the SMOD database were aggregated to form a “consultation”, the possibility that acts from two distinct consultations have been grouped together, or conversely, is a practicable scenario. However, the risk seems minor considering the number of variables used for the procedure (date, patient, health professional, place of dispensation, billing number) and would have no or minor consequences on the whole trajectory. A second limitation is related to the exclusion of consultations that may have taken place during an emergency or hospitalization episode. To avoid misrepresenting a succession relationship between a consultation and these two acute care services, we chose to exclude all acts occurring at the hospital for which the dates were matched with an emergency visit or a hospital stay registered in the emergency or hospitalization databases. This could have had the negative effect of excluding planned consultations with specialists if a planned medical consultation took place on the same day as an emergency visit or a hospital stay In the future, a clearer distinction between those consultations is necessary. However, there is, to date, no actual reliable variable to distinguish those consultations. Moreover, on the basis of the modelling process and data aggregation, we expect these limitations to have a relatively minor effect on the global 1-year trajectory. However, we noticed the generation of a potential artifact when modelling the trajectories of individuals between the consultation service and death. It appeared that death reports declared by physicians are registered in fee-for-service billing, often after an emergency visit or hospital stay, generating an artifact on the process map. This aspect was retained in this work to illustrate the impact of the data used to model trajectories. In further work, it will therefore be necessary to manually look for procedure codes corresponding to reports of death to exclude them.

Finally, the period of study had to be restricted to fit the CLSC database creation and matching ID implementation, which was effective in 2013 [43]. As a “recent” database, its validity remains to be demonstrated since it is less used for research and less regulated than the emergency or hospitalization databases are. Previous work performed in partnership with the INESSS Institute concerning homecare utilization in Quebec between 2013 and 2018 highlighted several limitations regarding the completeness of the database [31]. For example, according to the data, only 2% of the interventions involved physicians. Several physicians involved within local community service centers were consulted and advised that this could represent an important underestimation, potentially explained by the fact that physicians have no administrative obligation to enter interventions performed with patients within this database. Another example would be the latitude every provider disposes of to fill out the form about their interventions and the profile or nature of the interventions. Depending on the seniority and experience of each provider, a similar intervention could be registered in two different kinds of interventions, such as “palliative care” or “support for individuals with diminishing autonomy”. This variability can represent an issue to fairly represent the reality of homecare; however, as our trajectories were modeled at the macro level, including all interventions, this aspect is not considered an issue in this work.

#### Lack of palliative care information

An important limitation of the data used for this work is the lack of clear information about palliative care in the context of end-of-life trajectory modelling. A simple and concrete example is the fact that individuals could be in palliative care units during the last days of their life within a hospital, without any trace of it within the database if the hospital does not have a specific palliative care unit, which is fairly common. Notably, physicians providing palliative care services are often paid on a salary or mixed basis, as opposed to fee-for-service billing; hence, their services cannot be retrieved from the SMOD database. In addition, palliative homecare is often provided by physicians affiliated with the CLSC, who, as previously stated, have no administrative obligation to register their visits within the database. This could lead to the misleading message that physicians are not involved in end-of-life care and services or that few individuals can benefit from palliative care. Finally, the assignment of individuals to a “palliative care” profile is closely linked to health professionals’ expertise and to an administrative process imposed by the health system. A vital prognosis of three months is required in Quebec to receive palliative care, indirectly restricting access to individuals suffering from a terminal illness. Analyzing only administrative health data, without any expert judgment, would lead to the erroneous conclusion that few or no palliative care services are offered to individuals suffering from organic failure or physical and cognitive frailty. However, the reality on the ground is quite different. People suffering from organic insufficiency or physical and cognitive fragility can also be taken care of by CSLCs, often under the profile of “support for individuals with diminishing autonomy”. Further work is needed to better identify individuals who are able to benefit from palliative care within the administrative health data.

#### Process mining method

Finally, some limitations exist in the methodology, with the main limitation being the importance of the quality of the data provided in the event log, which follows the famous “garbage in, garbage out” principle. It is therefore necessary when applying this type of model to understand the data used and what it represents. This was demonstrated with the generation of the artifact of death certificates recorded in the physicians’ billing database, an element that had not been taken into consideration initially but that presents a very good example of the demonstration of an artifact. In addition, conversely, because some processes do not appear, they do not necessarily exist. This may be due to a lack of data in the event log or a lack of data due to a filter that is too restricted. Accordingly, another limitation is the choice of filter, as presented in the first section of the results. An overly restrictive filter, revealing only the core trajectory, could mask rare and relevant types of care use and result in too many isolated nodes. Conversely, a more permissive filter could generate a “spaghetti-like” process map, displaying an excessive number of edges and transition, thus providing insufficient information of quality. These subjective factors must be considered when applying process mining modelling, as they may lead to erroneous interpretations. Therefore, a clear understanding of the context and objectives is essential when applying process mining.

Another limitation would be the impossibility for the process maps to represent events that respond to a logic of parallelism, as in the graphical representation of the business process model and notation (BPMN) used for business process modelling and corresponding to the international standard ISO/IEC 19,510 [44]. Parallelism refers to a process for which, starting from an activity A, it is necessary to perform activities B and C, regardless of their order. Although the visual difference seems minimal, the issue is mainly the dynamics between activities A, B and C. In the context of process mining, the algorithm will distribute the individuals having performed A according to B or C in accordance with their first appearance in the event log and then indicate a mutual succession relationship between activities B and C, thus dividing the overall numbers or proportions. Unfortunately, this representation does not make it possible to see whether there are real differences in the way in which the activities are handled (B then C or C then B), which may be the result of organizational constraints or the saturation of certain services.

Finally, further work is needed to represent the temporality. Indeed, with the increase in knowledge concerning the end-of-life, it has been proven on numerous occasions that we have observed an intensification of health services during the last months, weeks and even days of life [30, 31, 45]. Unfortunately, as the process map currently presents the general typology of services used during the last year, including the succession relationships between all services, this intensification is not displayed. It would therefore be very relevant in the context of “trajectories” to add this notion of temporality. Further work is required in that regard. A potential short-term idea, which does not involve modifying the algorithm, would be to add the notion of temporality directly into the activity modalities. Thus, instead of presenting a single “emergency” activity, we can use the modalities “emergency T1”, “emergency T2”, “emergency T3” and “emergency T4”, with T1 to T4 representing the different quarters of the study year. This proposal, however, needs to be further evaluated in future work since it would have the disadvantage of making the map more tangled by adding both activities and edges. Methodological work aiming to modify the algorithm is underway and could represent an adequate solution to this issue.

## Conclusion

In general, despite the limitations associated with the data and certain visual limitations of the methodology, process mining seems to be a method that is both relevant and simple to implement. It provides a visual representation of the processes recorded in various health system databases and allows for the visualization of the different trajectories of healthcare utilization and their variation according to the sex of the individuals, their health status or their health region. When integrated into a Shiny or PowerBi-type dashboard, this methodology has the potential to provide a fairly comprehensive view of health service utilization. The advantage of integrating process mining into tools such as Shiny or PowerBi would be the ability to add stratification variables to display the desired process maps, as in the Ministry of Health and Social Services performance indicators.

## Electronic supplementary material

Below is the link to the electronic supplementary material.

Supplementary Material 1

## Data Availability

All the data can be obtained from the RAMQ and MSSS after a formal request to the ISQ.
