# Supplementary material for: Healthcare trajectories of aging individuals during their last year of life: application of process mining methods to administrative health databases
Source: BMC Med Inform Decis Mak. 2025 Feb 5;25:58. doi: 10.1186/s12911-025-02898-9 (PMC11796206; doi:10.1186/s12911-025-02898-9)
Supplement: Supplementary file 1 — Supplementary Material 1 [file 12911_2025_2898_MOESM1_ESM.pdf]

Supplemental 1 : Table of correspondence between ICD-10 codes and cause of death

ICD-10 code	Cause of death
C00-D48	Terminal disease
I231 ; I232 ; I233 ; I238 ; I25 ; I27 ; I42 ; I43 ; I50 ; I51 ; I520 ; I60 ; I61 ; I62 ; I63 ; I64 ; I67 ; I688 ; I69 ; I70 ; I73 ; I74 ; I792 ; I970 ; I971 ; I978 ; I980 ; I981 ; I988 ; J40 ; J41 ; J42 ; J43 ; J44 ; J47 ; J60 ; J61 ; J62 ; J66 ; J80 ; J841 ; J951 ; J952 ; J953 ; J96 ; J980 ; J981 ; J982 ; J983 ; J984 ; R060 ; R062 ; R063 ; R064 ; R065 ; R068 ; E10 ; E11 ; E12 ; E13 ; E14 ; E70 ; E71 ; E72 ; E75 ; E76 ; E77 ; E84 ; K44 ; K50 ; K51 ; K55 ; K56 ; K70 ; K71 ; K72 ; K73 ; K74 ; K75 ; K76 ; K77 ; K85 ; K86 ; K871" "K90 ; N02 ; N03 ; N04 ; N05 ; N11 ; N12 ; N136 ; N160 ; N18 ; N19 ; N25 ; N312 ; N318 ; N319 ; N82 ; M05 ; M06 ; M13 ; M15 ; M21 ; M30 ; M31 ; M32 ; M33 ; M34 ; M35 ; M41 ; M42 ; M43 ; M45 ; M46 ; M47 ; M48 ; M49 ; M50 ; M51 ; M53 ; M54 ; M638 ; M80 ; M81 ; M820 ; M821 ; M843 ; M844 ; M86 ; M87 ; M88 ; M907 ; M961 ; L305 ; L40 ; L41 ; L42 ; L440 ; L93 ; L945 ; A520 ; A521 ; A522 ; A523 ; A527 ; A810 ; A812 ; B15 ; B16 ; B17 ; B18 ; B19 ; B20 ; B21 ; B22 ; B23 ; B24 ; Q01 ; Q02 ; Q03 ; Q04 ; Q05 ; Q06 ; Q078 ; Q079 ; Q20 ; Q21 ; Q22 ; Q23 ; Q24 ; Q25 ; Q26 ; Q27 ; Q28 ; Q31 ; Q33 ; Q40 ; Q60 ; Q61 ; Q63 ; Q64 ; Q65 ; Q66 ; Q67 ; Q68 ; Q714 ; Q75 ; Q76 ; Q77 ; Q78 ; Q79 ; Q850 ; Q86 ; Q87 ; Q89 ; Q90 ; Q91 ; Q92 ; Q93 ; Q95 ; Q96 ; Q97 ; Q99 ; D60 ; D61	Organic failure
G03 ; G04 ; G05 ; G07 ; G10 ; G11 ; G12 ; G20 ; G21 ; G22 ; G23 ; G30 ; G31 ; G32 ; G35 ; G36 ; G37 ; G477 ; G518 ; G551 ; G608 ; G70 ; G71 ; G72 ; G73 ; G80 ; G81 ; G82 ; G83 ; G90 ; G91 ; G92 ; G93 ; G94 ; G95 ; G96 ; G97 ; G98 ; G99 ; F00 ; F01 ; F03 ; F05 ; F06	Frailty

Supplemental 2: Process maps of healthcare utilization in the last year of life for individuals aged 65 years and over in Quebec between 2013 and 2018. Full size

A) General unfiltered map (p. 3);

B) 10% filtered map, only edges followed by a minimum of 10% of the population are displayed (p.4);

C) For the 50% filtered map, only edges followed by a minimum of 50% of the population are displayed (p.5)

The node color and edge thickness are proportional to the proportion of individuals involved

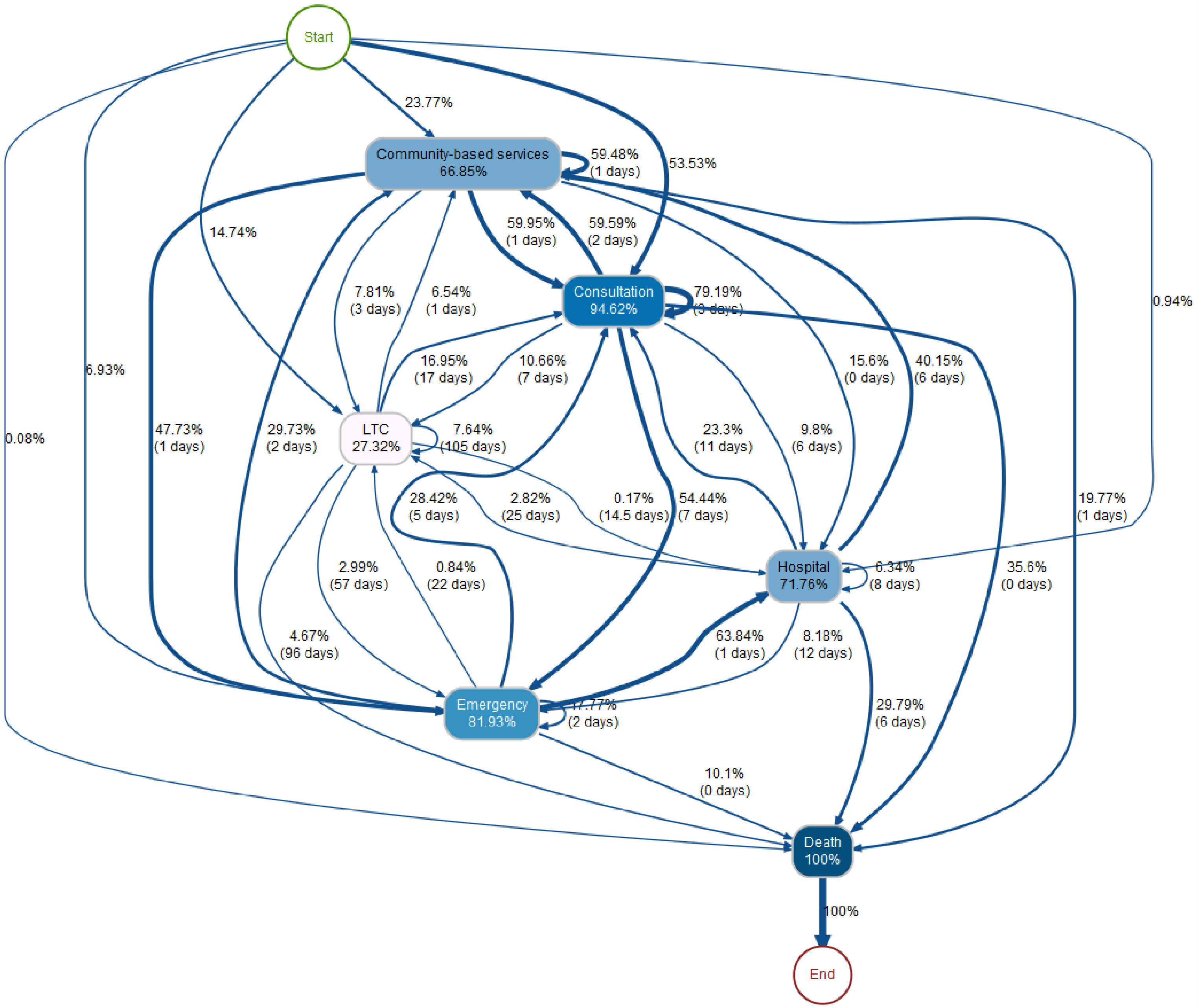
Start
23.77%
Community-based services
66.85%
59.48%
(1 days)
53.53%
14.74%
59.95%
(1 days)
59.59%
(2 days)
Consultation
94.62%
79.19%
(3 days)
0.94%
7.81%
(3 days)
6.54%
(1 days)
6.93%
16.95%
(17 days)
10.66%
(7 days)
15.6%
(0 days)
40.15%
(6 days)
0.08%
47.73%
(1 days)
29.73%
(2 days)
LTC
27.32%
7.64%
(105 days)
23.3%
(11 days)
9.8%
(6 days)
28.42%
(5 days)
2.82%
(25 days)
0.17%
(14.5 days)
54.44%
(7 days)
19.77%
(1 days)
2.99%
(57 days)
0.84%
(22 days)
Hospital
71.76%
6.34%
(8 days)
35.6%
(0 days)
4.67%
(96 days)
63.84%
(1 days)
8.18%
(12 days)
Emergency
81.93%
17.77%
(2 days)
29.79%
(6 days)
10.1%
(0 days)
Death
100%
100%
End

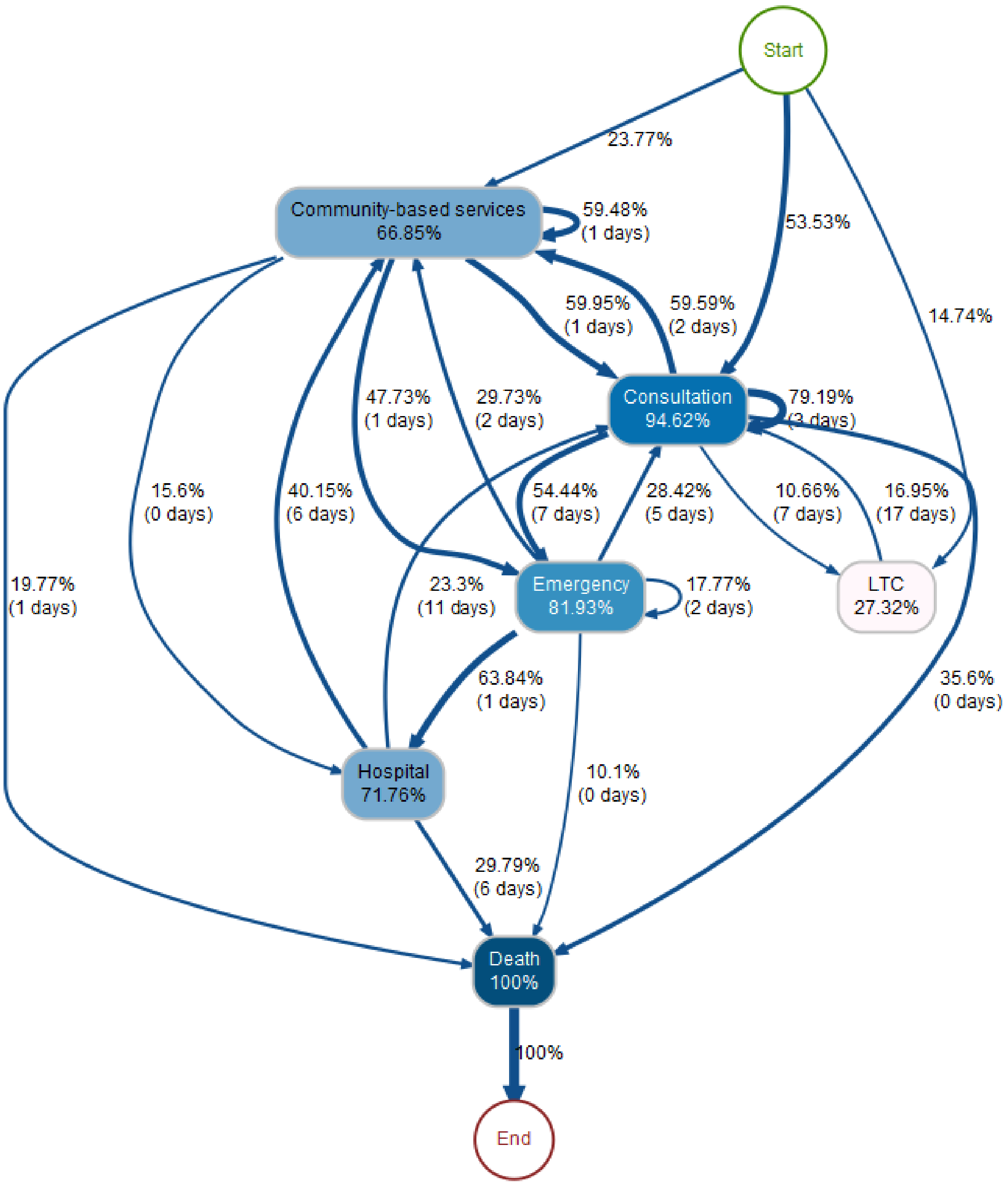
Start
23.77%
53.53%
14.74%
Community-based services
66.85%
59.48%
(1 days)
59.95%
(1 days)
59.59%
(2 days)
Consultation
94.62%
79.19%
(3 days)
47.73%
(1 days)
29.73%
(2 days)
15.6%
(0 days)
40.15%
(6 days)
54.44%
(7 days)
28.42%
(5 days)
10.66%
(7 days)
16.95%
(17 days)
19.77%
(1 days)
23.3%
(11 days)
Emergency
81.93%
17.77%
(2 days)
LTC
27.32%
63.84%
(1 days)
35.6%
(0 days)
Hospital
71.76%
10.1%
(0 days)
29.79%
(6 days)
Death
100%
100%
End

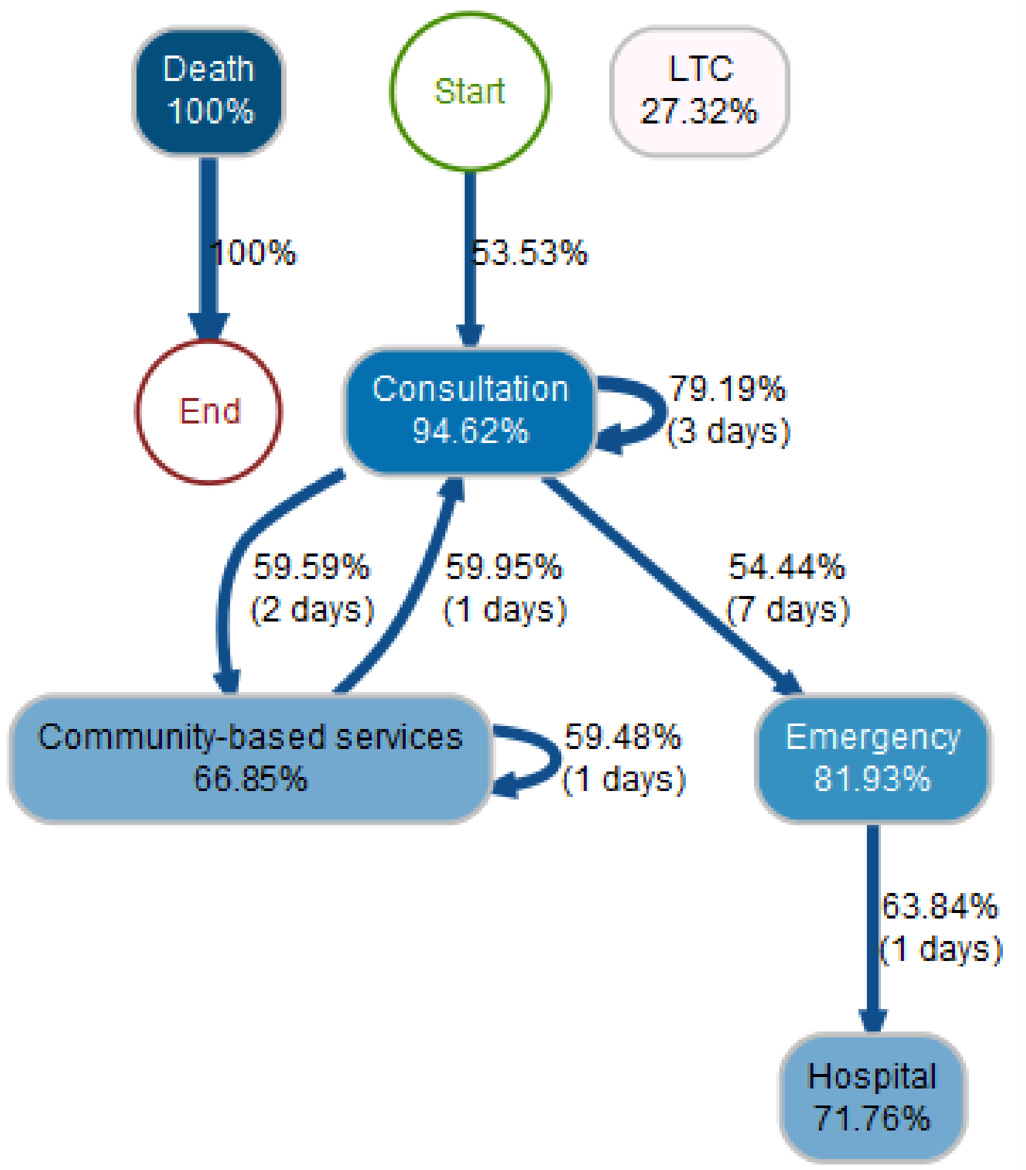
Death
100%
Start
LTC
27.32%
100%
53.53%
End
Consultation
94.62%
79.19%
(3 days)
59.59%
(2 days)
59.95%
(1 days)
54.44%
(7 days)
Community-based services
66.85%
59.48%
(1 days)
Emergency
81.93%
63.84%
(1 days)
Hospital
71.76%

Supplemental 3: Process maps of healthcare utilization in the last year of life for individuals aged 65 years and over in Quebec between 2013 and 2018 according to their cause of death. Full size

A) Terminal disease (p. 7);

B) Organic failure (p.8);

C) Physical or cognitive frailty (p.9);

D) Other or unknown (p.10)

The node color and edge thickness are proportional to the proportion of individuals involved

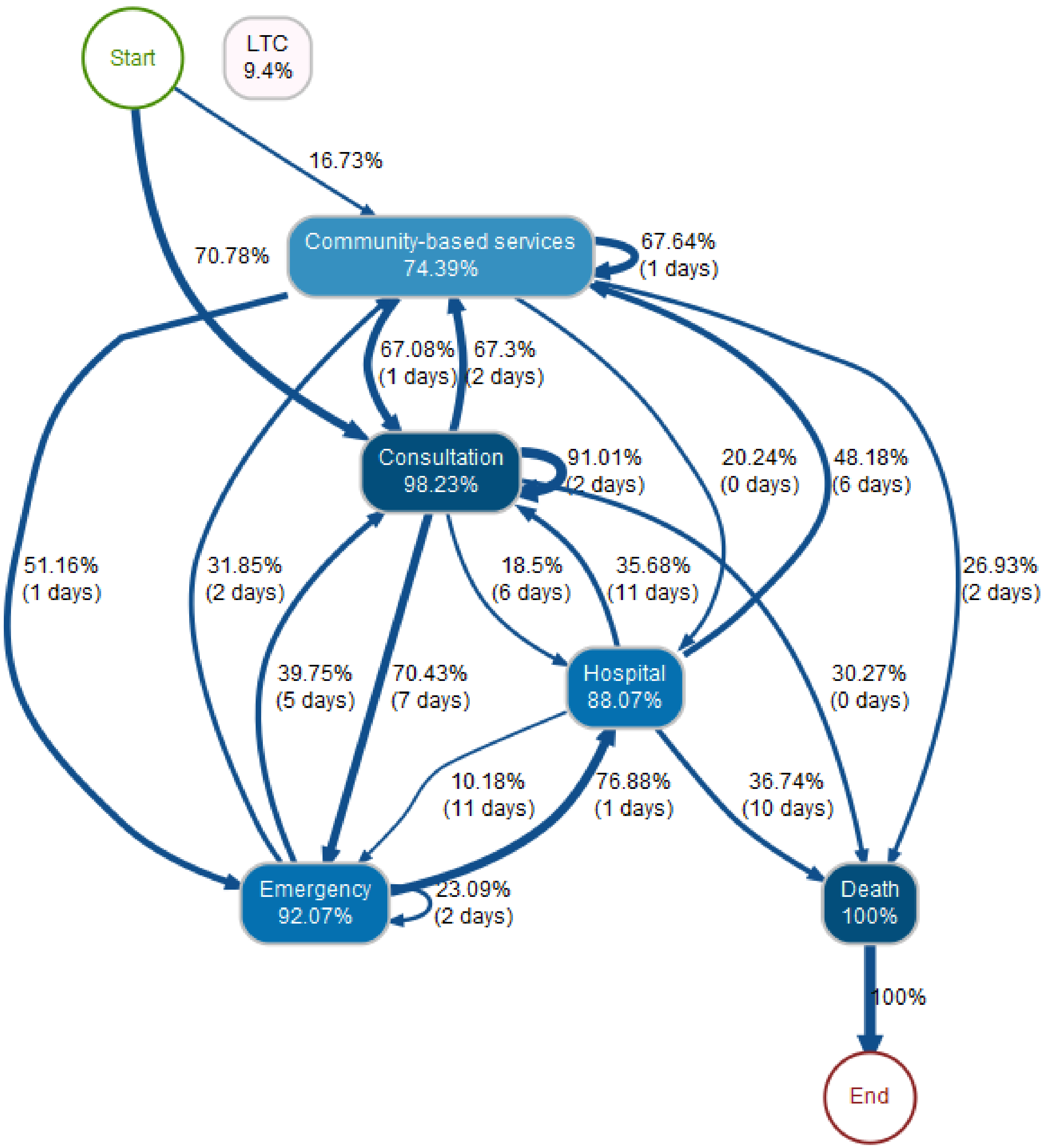
Start
LTC
9.4%
16.73%
70.78%
Community-based services
74.39%
67.64%
(1 days)
67.08%
(1 days)
67.3%
(2 days)
Consultation
98.23%
91.01%
(2 days)
20.24%
(0 days)
48.18%
(6 days)
51.16%
(1 days)
31.85%
(2 days)
18.5%
(6 days)
35.68%
(11 days)
26.93%
(2 days)
39.75%
(5 days)
70.43%
(7 days)
Hospital
88.07%
30.27%
(0 days)
10.18%
(11 days)
76.88%
(1 days)
36.74%
(10 days)
Emergency
92.07%
23.09%
(2 days)
Death
100%
100%
End

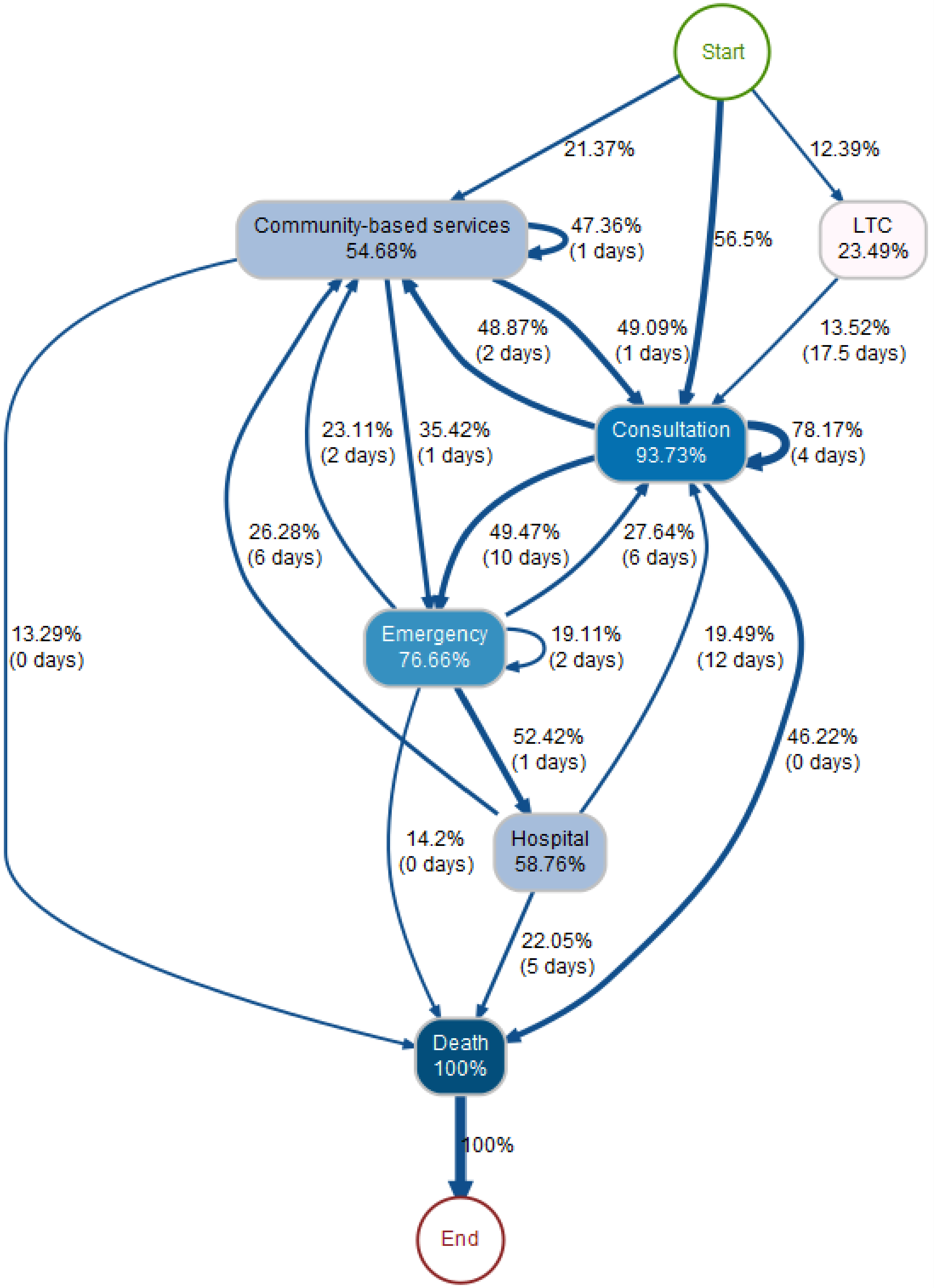
Start
21.37%
56.5%
12.39%
Community-based services
54.68%
47.36%
(1 days)
LTC
23.49%
48.87%
(2 days)
49.09%
(1 days)
13.52%
(17.5 days)
Consultation
93.73%
78.17%
(4 days)
23.11%
(2 days)
35.42%
(1 days)
26.28%
(6 days)
49.47%
(10 days)
27.64%
(6 days)
13.29%
(0 days)
Emergency
76.66%
19.11%
(2 days)
19.49%
(12 days)
52.42%
(1 days)
46.22%
(0 days)
14.2%
(0 days)
Hospital
58.76%
22.05%
(5 days)
Death
100%
100%
End

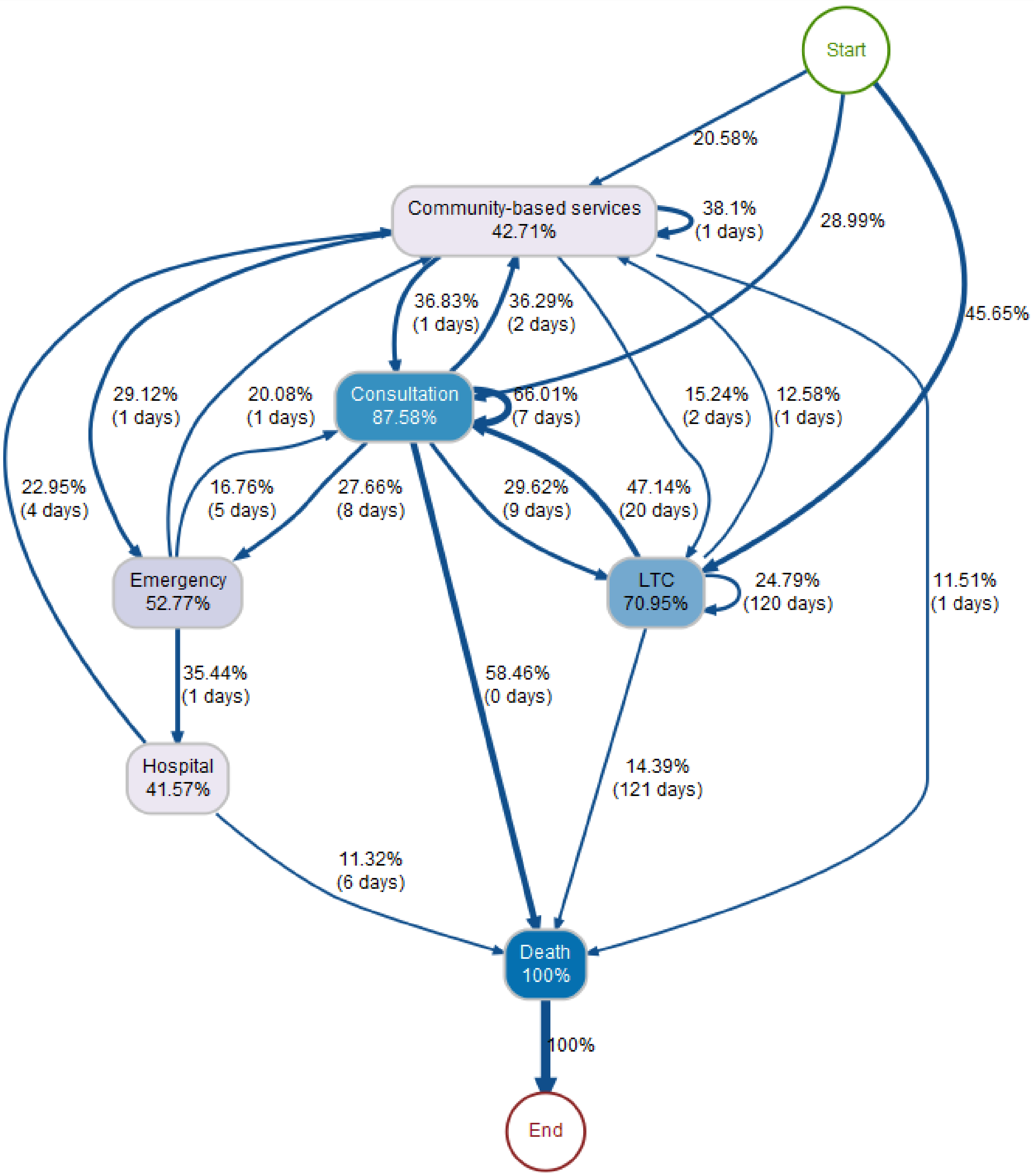
Start
20.58%
Community-based services
42.71%
38.1%
(1 days)
28.99%
45.65%
36.83%
(1 days)
36.29%
(2 days)
29.12%
(1 days)
20.08%
(1 days)
Consultation
87.58%
66.01%
(7 days)
15.24%
(2 days)
12.58%
(1 days)
22.95%
(4 days)
16.76%
(5 days)
27.66%
(8 days)
29.62%
(9 days)
47.14%
(20 days)
Emergency
52.77%
LTC
70.95%
24.79%
(120 days)
11.51%
(1 days)
35.44%
(1 days)
58.46%
(0 days)
Hospital
41.57%
14.39%
(121 days)
11.32%
(6 days)
Death
100%
100%
End

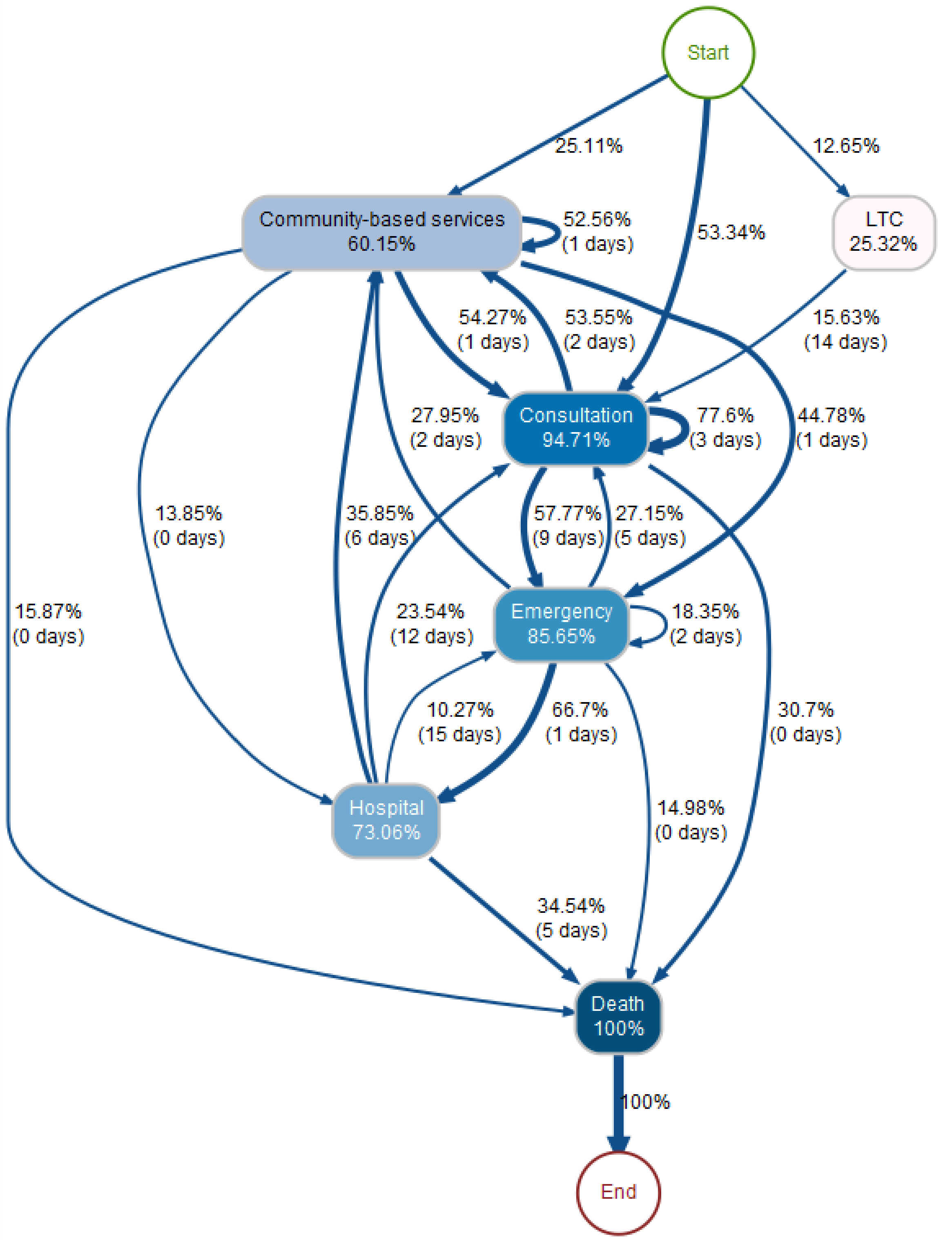
Start
25.11%
53.34%
12.65%
Community-based services
60.15%
52.56%
(1 days)
LTC
25.32%
54.27%
(1 days)
53.55%
(2 days)
15.63%
(14 days)
27.95%
(2 days)
Consultation
94.71%
77.6%
(3 days)
44.78%
(1 days)
13.85%
(0 days)
35.85%
(6 days)
57.77%
(9 days)
27.15%
(5 days)
15.87%
(0 days)
23.54%
(12 days)
Emergency
85.65%
18.35%
(2 days)
10.27%
(15 days)
66.7%
(1 days)
30.7%
(0 days)
Hospital
73.06%
14.98%
(0 days)
34.54%
(5 days)
Death
100%
100%
End
